# Cases of Fractures

**Published:** 1857-04

**Authors:** Frank Hastings Hamilton

**Affiliations:** Buffalo, N. Y.


### BUFFALO MEDICAL JOURNAL

AND

### MONTHLY REVIEW.

**VOL. 12.** APRIL, 1857. **NO. 11.**

### ORIGINAL COMMUNICATIONS.

ART. I. — *Cases of Fractures.* Reported by FRANK HASTINGS HAMILTON, M. D., Buffalo, N. Y.

(Concluded from Page 610.)

### TIBIA AND FIBULA.

#### LOWER THIRD.

CASE XC. — *Compound fracture. Result perfect.*

Walter Smith, of Pontoosac, Mass., æt. 9, broke his right leg near the ankle, in Oct., 1841. The fracture was compound.

I reduced the fracture and applied lateral splints, laying the limb upon its outer side. Three months after a fragment of bone exfoliated of the size of a twenty-five cent piece, and the wound then closed.

One year from this date the limb was sound, straight, and without any discoverable shortening.

Case XCI.— *Compound fracture. Result perfect.*

John Delaney, æt. 11. Oct. 25, 1855, his right leg was traversed by an empty railroad car, breaking both bones near the upper end of the lower third. The fracture was compound.

Dr. Lay and myself in attendance.

We found the fragments of the tibia very movable, but not much displaced. Having secured a carefully padded splint to the fibular side of the limb, we laid it upon a pillow, resting upon its outside and slightly flexed.

With unimportant modifications, this constituted the treatment to the close. Pretty extensive suppuration ensued, and a spontaneous opening occurred on the outside of the limb at an early day. Finally, however, the wounds healed, and bony union was consummated without either shortening or deformity. The bones were united in four weeks.

Case XCII.— *Compound fracture. Result perfect.*

Cornelius Ecker, of Nunda, N. Y., æt. 38. The wheel of a railroad car passed over his right leg, breaking both bones at the upper end of the lower third. Compound fracture. The integuments covering the heel were torn off at the same time. He was placed under the care of a surgeon in Nunda.

Jan. 1854, one year after the accident, he became an inmate of the Buffalo Hospital, for the treatment of an ulcer upon the heel, which had remained ever since the injury was received.

I found the bones of the leg united without shortening. The lower end of the upper fragment was displaced backward about three lines. I could not detect any ensheathing callus.

Case XCIII.— *Compound fracture. Result perfect.*

Wm. Harrington, æt. 32, broke both bones of the leg about three or four inches above the ankle. Fracture compound. Dr. Dieffendorf, of Herkimer Co., N. Y., was employed.

Twenty-two years after, the limb was perfect

Case XCIV.— *Compound fracture. Result imperfect.*

Henry Donegan, of Buffalo, adult. Fracture of tibia and fibula near the upper end of the lower third. At the Buffalo Hospital of the Sisters of Charity.

March 5, 1853, I removed all dressings. The lower end of the upper fragment is in front and to the fibular side of the lower. The limb is shortened one inch. Bony union was at this time complete, and there was no ensheathing callus which could be felt. I laid the limb on pillows.

CASE XCV.— *Compound fracture. Result imperfect.*

Wm. A. Hart, æt. 30, fractured the right leg three inches above the ankle. Tibia projected through the skin. Fracture of tibia oblique.

The fracture was treated by Dr. Walworth, of Fredonia, N. Y.

Twenty-nine years after the fracture occurred, the seat of fracture in both bones could be plainly traced, but I could not discover any displacement He assured me, however, that it was a little shortened; perhaps one-quarter of an inch.

The left leg had been broken fifteen years before, and it had always remained shorter than the right until after the right leg had been broken also, and they were now of the same length.

The ankle of this leg had occasionally been painful.

CASE XCVI.— *Compound fracture. Result imperfect.*

Edward Rubens, of Buffalo, æt. 14, broke his left leg, July 15, 1853, by jumping from a height of eight or ten feet, and striking on his feet.

Dr. Mixer was immediately called, and dressed the limb temporarily. On the same day I was, by Dr. Mixer's request, associated with him in the charge of the case.

I found the leg broken near the upper end of the lower third. The tibia was broken obliquely: opposite the seat of fracture, but on the inner side, was a wound about one inch in length, which was gaping, and through which the end of the upper fragment could be seen, denuded of its periosteum and roughened. It was still bleeding a little.

We laid the limb over a double inclined plane, and secured the foot so as to make extension and counter-extension as much as was practicable: applying at this time no lateral splints or other dressings. The limb was then covered with cloths moistened with cool water.

July 16, we substituted irrigations for the wet cloths. After a few days, the swelling having abated, lateral splints were applied, and still later, the paste bandage was substituted for the lateral splints.

Aug. 20th, we opened the paste bandage and found the fragments united, but the wound in the flesh had not entirely closed.

Sept. 2d, it is recorded in my notes that the wound was closed, the projection of the upper fragment could scarcely be discovered; the limb was straight, shortened one-quarter of an inch, and there was no ensheathing callus to be seen or felt.

One year later I found the shortening the same, but the limb did not appear so straight as at first, and the projection of the upper fragment in front of the lower was more distinct. The parents thought I permitted him to walk too soon, and that this had occasioned the change, but I think the change consisted in the disappearance of all of the fibrinous effusion which had concealed the deformity.

He walks without any halt or impediment.

CASE XCVII.— *Compound fracture. Result imperfect.*

J. O'Bryan, æt. 26. Fracture compound, and at a point about three or four inches above the ankle.

O'Bryan was treated at the Bellevue Hospital, New York. One small fragment of bone exfoliated.

Five months after the fracture occurred, I found the bones united and the limb shortened three-quarters of an inch.

CASE XCVIII.— *Compound fracture. Result imperfect.*

Pat. Burke, æt. 26, broke both bones of right leg near the upper end of the lower third, Oct. 18, 1850. Fracture compound.

Dr. Green, of Cattaraugus Co., N. Y., was employed. Patient was eleven weeks in bed.

March 16, 1851, five months after the accident, he was received into the Buffalo Hospital with an inflamed ulcer over the seat of fracture. Ulcer one inch in diameter. Leg shortened half an inch. Fragments united at an angle which is salient forward. The left leg is œdematous.

CASE XCIX.— *Compound fracture. Result imperfect.*

Michael Fellinger, æt. 24, fractured his left leg in April, 1852. Fracture compound: near the upper end of the lower third.

He was attended by a regular surgeon.

Three months after the accident, he was admitted to the Buffalo Hospital of the Sisters of Charity. The fragments were then united, with the lower end of the upper fragment projecting in front; the lower part of the leg, with the foot, falling backward. The skin is very tender and painful over the projecting bone, and threatens ulceration. He is in good health.

Case C.— *Compound fracture. Result imperfect.*

George W. Buffon, æt. 33, fractured both bones within three or four inches of the ankle. Fracture oblique and compound. I dressed the limb with side splints and laid it over Rose's double inclined plane, securing the foot to the foot board, and making as much extension as I could.

One year after the fracture, I found him at work, but limping badly in consequence of the swelling and tenderness which still remained about the ankle-joint. He said the leg was not shortened, but on measuring I found it shortened half an inch.

Case CI.— *Compound fracture. Result imperfect.*

A. Rogers, æt. 30, broke the right tibia and fibula, in 1846. Fracture at the upper end of the lower third. Compound. He was taken to the New Orleans Charity Hospital, and placed under the care of Dr. Stone.

Dec. 27, 1848, Rogers was admitted to the Buffalo Hospital of the Sisters of Charity, for the treatment of an intractable ulcer, situated over the point of fracture. He says he remained in bed at the New Orleans Hospital, two months, when the bones were united, and the wound in the soft parts was healed.

Six months ago he hurt the scar, and a ulcer was rapidly formed, which has never again been closed.

On measuring the leg I found it shortened by overlapping, half an inch. Manifest projection at seat of fracture and directly under the ulcer.

Feb. 12, 1849, he was dismissed with the ulcer nearly, but not entirely, healed.

Case CII.— *Compound fracture. Result imperfect.*

Willard Clark, æt. 14, of Evans, Erie Co., N. Y., fell under a loaded wagon, lacerating and breaking his right leg. The skin was removed from the leg quite extensively.

Dr. Aldrich, a very clever young surgeon, of Evans, had charge of the lad.

Aug. 16, 1848, two weeks after the accident, I was called by request of Dr. Aldrich. I found the limb suppurating freely, and resting upon a canvas very ingeniously suspended within a fracture box. I saw no occasion to make any change in the plan of treatment.

Seven years from this time the leg was shortened one inch, and the lost integument was not completely repaired. It was still discharging matter.

In 1856, a friend informed me that the sore had finally healed. He walks with a slight halt.

CASE CIII.— *Oblique, compound fracture. Result imperfect.*

Lewis Dodge, æt. 31. Right leg broken in 1831, by the fall of a piece of timber upon the top of the knee. Fracture at the upper end of the lower third, oblique and compound, the tibia projecting through the skin nearly an inch.

The fracture was reduced by Dr. Sydney H. Blossom, of Durhamville Oneida Co., N. Y., within five hours after its occurrence. Three or four efforts were made, and it required the strength of four men, to accomplish the reduction. During the first four days, Hutchinson's splint was employed, for the purpose of making extension and counter-extension; after which small side splints were substituted and a double inclined plane. This treatment was continued six weeks. An ulcer occurred on the heel from pressure against the splint.

I examined the limb fifteen years after the accident, and found it shortened half an inch, with a slight lateral displacement of the bone. He had himself supposed the limb not shortened. The limb was, in other respects, perfect.

CASE CIV.— *Compound fracture. Abscess of tibia and amputation after twelve years.*

Col. D., of Quincy, Chautauque Co., N. Y., æt. 40, was run over by a carriage, in 1844, breaking both bones of the left leg a little above the ankle. Dr. Stockton, of Pa., was employed. The fracture was compound. The fragments united in the usual time and with but little deformity, but the pain and swelling continued, and finally suppuration ensued. After many years of suffering, I amputated the leg near its middle, in Aug., 1856. I

found the tibia inflamed and expanded, from the joint to about five inches above, and containing an abscess large enough to hold three ounces of pus. There were two large openings leading from it to the surface, but it contained no dead bone. He now recovered rapidly.

CASE CV.— *Compound, comminuted fracture. Result imperfect.*

Christian Schaeffer, of Buffalo, æt. 32, was kicked by a horse, while sitting in his buggy, May 29, 1854, breaking his right leg four inches above the ankle. He was also immediately thrown from his carriage, and the wheels passed over the thigh of the same side, bruising it severely. A wound existed opposite the fracture of the tibia, which was evidently made by the cork of the horse's shoe, and this bone was so comminuted that I was able to remove with my fingers five or six small fragments, and other partially detached fragments were permitted to remain. The wound bled freely.

I dressed the wound with lint, and supported the limb with splints and junks on all sides, and laid it in a box. Subsequently I applied a paste bandage. Complete bony union was not effected until about twelve weeks after the accident. The loose fragments which I permitted to remain were not exfoliated. At no time in the progress of the case could I detect ensheathing callus. The wound was kept almost constantly covered and compressed by bandages and compresses, to which, in some measure, I attribute the favorable issue.

Aug. 22, 1856, nearly two years after the accident, I found the limb shortened half an inch: the upper fragment lying a little on the fibular side of the lower. His limb is as useful as before.

CASE CVI.— *Compound, comminuted fracture. Result imperfect. Prosecution. Successful defence.*

Christian Shoemaker, æt. 50. Compound, comminuted fracture, produced by the fall of a piece of timber upon it. Right leg. Three or four inches above the ankle.

Fracture of tibia very oblique, so that a sharp point projected about one-eighth of an inch. Another small wound was made by another small fragment, which was not completely detached from the soft parts.

He had had indolent ulcers on the same leg for two years.

Dr. Jeyte, of Buffalo, saw and dressed the fracture within two hours. He placed the limb in Sutter's swing splint, and applied cold water dressings.

Extension and counter-extension were made from the extremities of the splint. On the seventh day pasteboard splints were applied, and the limb was again laid in Sutter's splint. The bandages were now permitted to remain undisturbed two or three weeks, the limb resting constantly on its back upon a sacking.

When united the limb was found to be shortened. There was an ulcer on the back of the heel, and the limb was considerably bent at the seat of fracture, so that the foot and lower part of the leg fell backward.

Dr. Jeyte was prosscuted in this case for malpractice, but no verdict was obtained against him; so I am informed.

CASE CVII.— *Compound, comminuted fracture. Result imperfect.*

John B. Foot, æt. 39, fractured both bones of the left leg, about two inches above the ankle-joint. Fracture compound and comminuted. Produced by being thrown from his carriage when his horses were running, his leg being caught in the wheel.

Dr. Bissel, of Geneseo, N. Y., was employed. The contusion was so great that it was found impossible to use extension, or to apply anything but the most simple dressings. The limb was laid in a box and carefully supported. Several fragments of bone were loosened and detached, including, finally, the malleolus internus of the tibia. His recovery was slow and tedious.

I examined the limb six years after the accident occurred, and found it shortened one inch and quite crooked. It is still, at times, painful, and especially when he uses it much.

CASE CVIII.— *Compound, comminuted fracture. Amputation and recovery.*

Arthur Prime, of Buffalo, aged about 30 years, was crushed, Oct. 15th, 1852, between a boat and the dock, breaking his right leg near the upper end of the lower third. Fracture compound and comminuted.

Drs. Baker and Lockwood were called. They found the limb bleeding freely from the wound in front of the tibia, and applied a bandage snugly from the ankle to the knee, with a compress over the wound.

Oct. 16, I was requested to see the patient with Drs. Baker and Lockwood. Notwithstanding the bandage and compress, the bleeding continued. The foot was much swollen. We removed the bandage and enlarged the wound freely so as to search for the artery, but could not find it. I removed one fragment of bone and found several others quite loose. Without delay, assisted by Drs. White, Baker and Lockwood, I proceeded to amputate the limb a little below the knee; the patient being under the influence of chloroform.

On examination of the limb we found the fibula broken into six fragments, and the tibia into four, and their lower extremities separated from each other. Neither the anterior, nor posterior tibial arteries were ruptured, nor the fibular, yet the areolar tissue between the muscles, and underneath the skin, from the knee down, was filled with blood. The skin and muscles were also very much bruised and lacerated.

Sloughing of a portion of the flap, with secondary hæmorrhage, subsequently occurred, but he finally recovered.

Case CIX.— *Compound, comminuted fracture. Both legs. Result imperfect.*

John Curran, æt. 23. Left leg broken near the ankle. (See Case XLI, middle third.)

Case CX.— *Compound, comminuted fracture, complicated with other severe injuries. Result imperfect. Resection of projecting bone.*

July 26, 1853, James A. Manley, æt. 30, was caught in the machinery of a steam engine, breaking his right leg at the upper end of the lower third. The wound in the skin opposite the fracture, was three-quarters of an inch in length, and a small fragment of the tibia could be felt loose within. His skull was also fractured near the superior angle of the occipital bone, and a large wound in the scalp comminuted with this fracture. After the injury, he remained for some time unconscious.

Drs. Strong and Hawley were called, and subsequently I was added to the council. We found his consciousness returning, and as the skull was only very little depressed at the point of fracture, we did not trephine, but dressed the wound with simple cerate.

The fragments of the tibia overlapped, and we were unable to replace them completely. The leg was also so much bruised and lacerated that we hesitated whether we ought to make any attempt to save it. It being determined, however, that the attempt should be made, we dressed the wound and supported the limb with well cushioned splints, with moderate bandages, and laid it upon a pillow. Cool water irrigations were then directed to be made constantly.

Aug. 5th, we dressed the limb with a paste bandage, and laid it over a double inclined plane.

Aug. 25th, we found the tibia projecting at the wound, and having placed him under the influence of chloroform, I removed, with a chain saw, about half an inch of the bone. We were still, however, unable to make the bone resume its place completely, although it was sufficiently shortened; yet it no longer projected beyond the skin. The consolidation of the bones and the closure of the wound now went on rapidly, and it has, finally, been cured, with a shortening of half an inch. The lower end of the upper fragment projects considerably in front, but, at the end of a year, the limb seems as strong and as useful as ever.

I especially remarked in this case the value of the paste bandage in diminishing the swelling. Although it was applied over suppurating wounds, and the limb was completely encased for several days, yet by its uniform and steady support it gave great relief to the patient, and very much diminished the swelling.

Case CXI.— *Compound fracture. Resulting in amputation on the fifteenth day.*

Mark Sullivan, æt. 40; at the Buffalo Charity Hospital. Fracture near the upper end of the lower third of the leg. Compound. An attempt was made to save the limb, but amputation became necessary on the fifteenth day. The patient recovered.

Case CXII.— *Compound, comminuted fracture, complicated with severe contusion, &c. Amputation on the thirty-fourth day and recovery.*

Mary, daughter of John Gibbons, æt. 5, was caught under the rollers upon which a small frame house was being moved, July 16, 1853, crushing one of her legs.

Dr. Mixer, of Buffalo, was called. On the following day I was requested to see the case with him. The foot and leg were greatly swollen and very much discolored, and in addition to the severe injury done to the foot, the leg was broken near its lower end.

No splints were applied. For a time we entertained some hope of saving the entire limb, but eventually the foot sloughed off, and also the skin of the leg to within four inches of the knee.

Aug. 19, 1853, assisted by Drs. Mixer and Boardman, I amputated just below the knee. Her recovery has been complete.

CASE CXIII.— *Compound, complicated fracture. Amputation. Recovery.*

Alice Thompson, æt. 35. April 19, 1853, her leg was crushed under a train of cars. She was immediately brought to the Buffalo Hospital, and within two hours of the time at which the accident occurred, I amputated the leg near its middle. She recovered after several weeks.

CASE CXIV.— *Compound, comminuted fracture. Excision of the protruding bone, on the nineteenth day. Result imperfect. Prosecution and successful defence.*

Having no recorded notes of this case, although it came under my own observation, I shall copy a report of the trial of the defendants for alleged malpractice, which appeared, some days after the dismissal of the case, in one of the Cortland Co. papers. I shall omit only such portions of the report as may have a personal reference to parties not directly implicated in the suit.

"Wm. Smith *vs.* Miles Goodyear and Frederick Hyde.

"This suit was tried at the recent Cortland Circuit. It was one of unusual interest, not only to the public, but to the medical profession generally. It was brought against the defendants for alleged malpractice, as physicians and surgeons. The damages were laid at two thousand dollars.

"The high standing of the defendants—the long and faithful professional toil of Doct. Goodyear—the bright promise of the opening course of Doct. Hyde—render the case of deep importance to them.

"The prominent facts brought out on the trial, are as follows:

"The plaintiff, on the 4th of July, 1839, fell from the stage of a building, in the town of Cortlandville, and broke his left leg, just above the ankle. The bone was thrust through a thick boot leg, and came in contact with the ground, being what is termed in surgery, a compound, comminuted fracture. It was set and dressed by Doct. A. B. Shipman. The plaintiff was nearly fifty years of age, and had been for a long time grossly intemperate. The weather at the time, was extremely warm. On the 5th of July he was removed to the county almshouse. The superintendent, having previously employed Messrs. Goodyear and Hyde, (who were partners,) to attend to the sick inmates, the plaintiff became their patient. They placed the limb upon a double inclined plane, confined it with splints, and applied light dressings. He was placed in the care of a nurse by the name of Samuel Baker. He was attended, and the limb dressed, every day, by the defendants, except on one occasion, when Doct. F. T. Maybury, who was then a student in the office of the defendants, dressed it in their stead. The wound was about three and a-half inches long, and about two inches wide. It soon became highly inflamed and the limb swollen. The weather continued warm, and the flesh about the wound sloughed off, and left the bone naked, the end of which was dead, in consequence, as was proved, of the violence of the blow, at the time of the fracture. The defendants continued their attendance until the 13th of July, the ninth day after the fracture, when they were induced to believe, from the state of the weather, the age and habits of the patient, and apprehensions of fever, that it was a proper case for amputation. The plaintiff was averse to it and it was not done. The defendants continued, as usual, their daily dressings of the limb, in the effort to save it whole, until the 23d of July, when, at the plaintiff's request, they were relieved from the charge of it. He sent for Dr. Shipman, who, (deeming that the case required it,) on the 23d of July, proceeded to saw off about one inch from the end of the bone. He with others, from that time, continued to attend on the plaintiff.

"It was alleged, that the defendants were guilty of malpractice in neglecting to dress the limb; and second, in not sawing off the end of the bone, it having lost its vitality.

"The case wore a very grim visage at its opening to the jury. The counsel for the plaintiff, M. B. Butterfield, Esq., had drawn to his aid the skill and talents of L. A. Spencer, Esq., of Utica. The counsel for the defendants, H. Ballard, Esq., had associated with him the forensic powers and experience of B. D. Noxon, Esq., of Syracuse. But, as often happens, 'the success of the war, did not come up to the sounding phrase of the manifesto.'

"The charge for neglecting to dress the limb, was met and overthrown, by Samuel Baker, the nurse, who was introduced by the plaintiff himself, and whose clear and intelligent testimony came with convincing power over the minds of every hearer, and scattered to the wind the base calumnies that had been industriously propagated over the country against the defendants. It was shown conclusively, that the patient was visited every day and the limb dressed.

"The great question agitated upon the trial, was as to the correctness *of the treatment* of the limb, by the defendants. The defendants had procured the attendance of a number of the most eminent surgeons in the State: James Webster, professor of anatomy, and Frank H. Hamilton, professor of surgery, in the Geneva Medical College; Docts. Mitchell, of Norwich; Hawley and Webster, of Ithaca; Miller and Smith, of Truxton; Bradford and Webster, of Homer; Burdick, of Preble; Bronson, of Virgil; Briggs, of Dryden; Benton, of Tioga; Maybury, of Freetown, and others. Messrs. Goodman and Hyde knew that they had been most wantonly assailed. They feared not to call to the stand these distinguished surgeons, to testify before the jury and the country as to the correctness of their practice. Most triumphantly were they sustained.

"Prof. Hamilton examined the leg in the presence of Doct. Hawley, the day the trial commenced; found it about one and a quarter inches shorter than the other, and about two inches larger around; with an open ulcer; and ascertained that the probe penetrated the bone about one inch.

"The evidence was proceeding on the part of the defendants, showing conclusively that their *practice*, as well as their *opinions*, throughout their connection with the case, was entirely right: when the counsel for the plaintiff, Mr. Spencer, impressed with the fullness and completeness of the defence, arose, and after paying a high compliment to the professional skill of Messrs. Goodyear and Hyde, voluntarily withdrew the suit.

"Seldom have we seen so deep a sensation manifested as on this occasion. The trial lasted a portion of two days, and the interest increased as the trial progressed. A mingled feeling of pleasure and indignation was felt by a large number in attendance, at the result, pleased to witness the triumphant vindication of professional merit and private worth; but indignant at *the foul spirit* that instigated the groundless prosecution.

"Messrs. Goodyear and Hyde had been pursued by a shameless tissue of falsehood for almost two years. The public mind was loaded with the sad story of the plaintiff's wrongs. Justice loudly demanded an investigation into the facts. It has been had, and the defendants have come out of it, with a reputation of fidelity, judgment and skill, made brighter by the test. Doct. Goodyear has resided in Cortland village over twenty years. Has reared his family here. From the beginning of his professional course, up to the present hour, his life has been distinguished for faithful, disinterested and laborious service.

"It has given him a hold on the affections of this people, which grows stronger by the lapse of time. Vain are all secret machinations against such a man. They will return to plague the inventer.

"Doct. Hyde may well congratulate himself on the happening of this event; for it has raised him in the estimation, not only of this community, but of the talented physicians and surgeons present at the trial. He is a young man thoroughly read in his profession. His deportment in the domestic circle to which his practice introduces him, is remarkable for dignity and decorum. The manner in which he passed through his trial is a true omen of his future success.

"Judge Mosely expressed himself highly gratified at the result of the trial, and in the course of his well timed remarks, spoke in warm praise of the professional foresight and judgment of Messrs. Goodyear and Hyde, as was shown in the case."

The plaintiff subsequently became an inmate of the Auburn State Prison, and, at the last accounts, some years later, the limb remained diseased and in a condition to justify the opinion of Drs. Hyde and Goodyear, that amputation would have been proper.

CASE CXV.—*Simple fracture of the fibula and malleolus internus, complicated with a dislocation of the tibia. Result imperfect.*

Jonathan Baker, æt. 33, of Castleton, Columbia Co., N. Y., was struck by a falling tree upon his right leg, breaking the fibula about four inches above its lower end, and carrying away, at the same time, the malleolus internus of the tibia. The tibia was dislocated also.

Drs. Peter and Abraham Hogaboom, of Columbia Co., had charge of the limb.

Thirty years after the occurrence of the fracture, Aug. 21, 1852, I examined the limb. It is neither shortened, or deformed, but it is emaciated and not as strong as the other. It is also quite tender about the ankle, and in this respect it is worse now than it was a year or so after the accident occurred.

CASE CXVI.—*Simple fracture of malleolus internus, and fracture of the fibula, with dislocation of tibia inward. Resulting in great deformity. Prosecution, &c.*

"—— Brockway, of Cortland, N. Y., aged about 27 years. Dr. A. B.

Shipman in attendance. The dislocation was never reduced, although repeated efforts were made to accomplish its reduction.

After one year, the patient called on me to know whether it could then be reduced. The lower end of the tibia projected inward about one inch, and the foot was slightly turned out. The fibula was bent inward against the tibia at the seat of fracture, four inches above its lower end. The motions of the joint were very limited. A prosecution was threatened, but after I had shown the patient a cast of one of my own cases, in the same position, he assured me that he was fully satisfied, and would not attempt to prosecute a claim for damages.

It would appear, however, from a statement made in the Boston Medical and Surgical Journal, vol. 31, pp. 140–2, that the case was tried in the Supreme Court of Cortland Co., N. Y., in May, 1844, and that the jury could not agree upon a verdict.

Case CXVII. — *Simple fracture of the fibula and of the malleolus internus, with dislocation of tibia inward. Result imperfect.*

I. Borland, of Borland's Mills, Erie Co., N. Y., aged 31 years. Mr. B. fell under a rolling log and dislocated his left tibia inward, breaking off the internal malleolus. The fibula was also broken, about four inches from its lower end. Dr. Sweetland, an old and experienced practitioner, was soon in attendance, who, with another surgeon, failed, after repeated efforts, to reduce the dislocation.

I saw Mr. B. twenty-four hours after the accident, and immediately proceeded to attempt the reduction. The foot and ankle were somewhat swollen, but not very greatly. The skin over the malleolus internus was discolored. The lower end of the tibia projected inward about one inch. We first flexed the leg upon the thigh and made extension with our hands during several minutes, constantly flexing and extending the foot upon the leg, and the leg upon the thigh, and in every possible way varying the position of the limb. To this mode of procedure we at length added forcible pressure upon the inside of the tibia near its lower end. We then placed the leg and thigh over a double inclined plane, and securing the limb firmly, we attached a screw to the foot, through a sandal and gaiter, and while the leg was flexed upon the thigh we renewed the extension. This was continued more or less during half an hour, and meanwhile pressure was made occasionally upon the inside of the tibia, and the position of the foot and leg, was, from time to time, slightly varied. The power which we were thus able to apply was very great, yet it did not for one moment produce any sensible impression upon the displaced bone. We continued our efforts for more than an hour, until we were satisfied that the reduction could not be accomplished, and until the patient begged us to desist. Anæsthetics were not then in use, and I am not certain whether Mr. B. was bled or not; but I doubt whether any such means would have increased our chances of success.

Four weeks from this date, Mr. B. walked on crutches; about one year he walked with a cane, and from that time, a period of eight years, he has walked without any support. For a long time he felt a yielding in his ankle, as the weight of his body settled upon this limb, but this gradually ceased, and now he walks without any halt, and as firmly as before the accident.

The tibia still projects inward about one inch; the foot inclines slightly outward, and the broken ends of the fibula rest against the tibia, and are there united.

Case CXVIII.—*Comminuted fracture, complicated with partial dislocation of the tibia. Result imperfect.*

Bernard Duffie, æt. 32. June 5, 1854, a very heavy stone fell upon his back and crushed him forward, breaking the left innominatum, (see fractures of innominata, Case VI,) and the right leg. The malleolus internus of the tibia was broken off and comminuted. The fibula was broken about two inches above the ankle. The tibia was also slightly displaced inward. I dressed the limb temporarily with a pillow and side splints, and sent him to the hospital.

Oct. 1, 1854, at the commencement of my service at the hospital, I found the tibia displaced forward and inward upon its articulation. The fibula was united with its broken ends resting against the tibia. He had recovered considerable motion in the ankle-joint. An ulcer, which had formed on the back of the heel, was closing up kindly.

Case CXIX.—*Simple fracture of the fibula and of the malleolus internus; with dislocation of the tibia inward. Result imperfect.*

Mrs. Elizabeth Swartz, of Buffalo, aged 59 years, fell six feet, striking upon the sole of her right foot, fracturing the fibula three inches above the ankle, and at the same time breaking the malleolus internus. The tibia was dislocated inward.

Dr. Hackstein reduced the dislocation, and dressed the leg with Dupuytren's splint. I saw Mrs. S. six weeks after the accident, by request of Dr. Hackstein, and again seven months after. The foot was then slightly turned out, the malleolus internus quite prominent, and the malleolar fragment depressed. The fibula had united with its broken ends resting against the tibia. The motions of the joint were embarrassed, and the ankle was still tender. She complained of her surgeon, and refused to pay his bill, but became satisfied when, on the same day, I sent to her one of my own patients, in whom the same accident had left a greater deformity.

Case CXX.—*Simple fracture of the fibula and of the malleolus internus of the tibia. Result perfect.*

John Flinn, was hit violently on his leg, Oct. 5, 1854, breaking the fibula a little above the ankle, and seperating the malleolus internus from the tibia.

The limb was dressed temporarily and very badly, by an empiric, but on the following day Flinn was received into the Buffalo Hospital. The leg was at that time a good deal swollen, and one of the splints was pressing very uncomfortably upon the external malleolus.

I removed all dressings, and finding the fragments were in place, and I presume they were never displaced; I laid the limb upon a pillow, with a couple of straight, board splints outside of the pillow, and supported the whole with tapes.

Oct. 7th, not being able to discover any motion or crepitus, I removed the side splints entirely, and simply laid the limb on its side upon a pillow.

Oct. 12, discovered a feeble crepitus, and applied a carefully moulded gutta percha splint. Laid the limb on a pillow, resting on the back of the leg and heel.

Oct. 26, discovered an ulcer on the heel. He had hitherto made no complaint of soreness in the part. Dressed ulcer with ung. basilicon. Fragments united. No shortening or deformity.

Case CXXI.—*Simple fracture of the malleolus internus and of fibula. Result perfect.*

W. S. Mooney, æt. 35. Both legs broken in a threshing-machine. (See Case XXXI.) Treated upon a double inclined plane.

Examined one year after. No defect in the joint, nor appearance of deformity.

CASE CXXII.—*Simple fracture of malleolus internus and of fibula. Result uncertain.*

Nicholas Fleek, Centerport, N. Y., æt. 51, a canaller, called upon me, May 31, 1853, with a fracture of the malleolus internus, and also a fracture of the fibula, three inches above its lower end. The accident had occurred some days before by the fall upon it of a barrel of flour, the chime striking upon the outside of the ankle. The foot was not dislocated. The upper end of the lower fragment of the fibula was inclined against the tibia, and I could not replace it.

I dressed the limb with three straight splints, well supported with junks. I have never seen or heard from him since.

CASE CXXIII.—*Simple fracture of malleolus internus, and of malleolus externus. Result uncertain.*

John McDonough, aged about 35, broke both malledi of the left leg, Nov. 2, 1852.

I was called, and applied a well padded splint of gutta percha to the back of the limb, merely as a means of support in moving the limb.

The fragments united, and I need not say that there was no shortening of the limb, but how long it was before the ankle regained its free motion, I am not able to say.

CASE CXXIV.—*Fracture of the malleolus internus, and also of the fibula, two inches above the ankle, with partial displacement of the tibia inward. Result uncertain.*

—— Ried, of Buffalo, æt. 7, fell from a fence, April 30, 1854. Dr. Leech and myself in attendance. The displacement of the tibia upon its articulation had been reduced before I saw him. The malleolus internus was broken off, and the fibula was broken two inches above its lower end.

We applied a splint and suitable compressors, with bandages.

May 29th he was walking on crutches. I have neither seen nor heard from him since.

CASE CXXV. — *Fracture of the malleolus internus, and also of the fibula four inches above the ankle, with dislocation of the tibia inward. Result imperfect.*

John Kerner, æt. 50, fell from a scaffolding, Oct. 14, 1853, striking upon his feet, dislocating the right tibia inward, and fracturing, at the same time, the malleolus internus and the fibula. The fibula was broken about four inches above its lower end. Dr. Lockwood was immediately in attendance, and having reduced the dislocation, he applied Dupuytren's splint. The next day, at the request of Dr. Lockwood, I took charge of the patient, assisted by Dr. Pupikofer. In a few days, vesications appeared over the malleolus internus, and we were apprehensive of sloughing. It was in part due to the splint, and in part to the protrusion of the bone in this direction, at the time of the accident. We took great pains afterward to avoid pressure at this point, and throughout the treatment we were extraordinarily attentive and careful.

On the sixth week we removed the dressings, and there was little or no deformity *apparent* upon either side of the limb. But, after the lapse of several months, when the swelling had entirely subsided, which at first (though slight) concealed the defects, considerable deformity was disclosed.

At the end of eighteen months, the tibia projects inward, and the malleolus is carried a little downward. The fibula leans against the tibia at the seat of fracture, and the foot inclines outward. There is also less freedom of motion in the joint than before the accident. He walks with a slight halt, but his ankle is not tender or painful.

CASE CXXVI. — *Simple fracture of the malleolus internus, and of the fibula, three inches above the ankle. Result imperfect.*

Mrs. Elizabeth Swartz, of Buffalo, æt. 59, fell, in May, 1854, and broke her right leg. She fell about six feet, striking square upon the bottom of her right foot.

Dr. Hackstein was called, and found the malleolus internus broken off, and the fibula broken three inches above its lower end. He dressed the limb with two side splints, with junks, etc.

Forty-six days after the accident occurred, I was requested to visit the patient in consultation with Dr. H.

The bones were united; the lower fragment of the tibia (malleolus internus) being slightly displaced toward the ankle-joint, and the fragments of the fibula resting against the tibia. The ankle-joint was stiff, and the heel was drawn up, throwing the toes downward. Ensheathing callus could be distinctly felt on the broken fibula, but none on the tibia.

Feb. 11, 1855, nine months after the accident, she called at my office. She was then able to move the ankle-joint pretty freely. In other respects the limb was as in my previous examination.

Case CXXVII.—*Simple fracture of malleolus internus, and of the fibula, three and a-half inches above its lower end. Result perfect.*

Mrs. John Murray, of Buffalo, æt. 37, slipped and fell on an icy board, March 15, 1855. Dr. Hill, her family physician, being called, requested me to be sent for and to take charge of the case.

I found the malleolus internus broken off and slightly depressed toward the ankle-joint. The fibula was broken three and a-half inches above its lower end. This I was able to determine only by pressing on the lower end of the fibula, by which a slight motion was communicated to the upper end of the fracture. There was no crepitus, yet Dr. Hill said that he felt crepitus distinctly when he first arrived. The ankle was already considerably swollen. Mrs. M., who weighed 192lbs, says that she felt a distinct snap as she fell, and before she struck the ground, and that on attempting to stand up her foot immediately turned out and she felt a second snap. She was suffering great pain.

I applied no splints, but only laid the limb on pillows and directed warm fomentations. The pains becoming more intense, I substituted, after a few hours, cool lotions for the warm fomentations. This soon gave her relief.

March 23, I applied Dupuytren's splint, securing it with a paste bandage.

April 3, I removed all dressings. Union of fragments complete. No en sheathing callus. I cannot discover that the malleolus internus is displaced. The upper fragment of the fibula is thrown toward the tibia.

After a few weeks her recovery was complete.

NOT BROKEN IN THE SAME DIVISIONS.

Case CXXVIII.—*Simple fracture of the tibia through its upper third, and of the fibula through its lower third. Result perfect.*

John Williams, æt. 23. Dec. 31, 1853, a load of wood fell upon his right leg, breaking the tibia through its upper third, and the fibula through its lower third. Fracture simple.

Dressed and treated by Dr. Charles Wilcox, of Buffalo, until Jan. 11, 1854, when he came under my care at the Buffalo Hospital. Dr. Wilcox dressed the limb on the first day, with pasteboard splints and rollers; and again renewed the dressings on the 7th of Jan. I removed the dressings on the 11th, and finding the fragments in place I reäpplied the same. About the 20th of Jan. I substituted a paste bandage.

Feb. 11, a little more than seven weeks after the accident occurred, I removed all dressings, and found the fragments united without ensheathing callus, and without shortening. Very slight projection of the tibia at the point of fracture.

Case CXXIX.— *Compound fracture. Tibia broken obliquely at upper end of lower third; and fibula about two inches below the upper end, (upper third.) Result imperfect.*

B. G. McKay, of Griffin's Mills, æt. 33, broke his right leg, April 5, 1851, by bracing his feet against the front of his carriage when his horses were descending a hill rapidly.

Drs. Williams, Needham and Allen, all excellent practical surgeons, were present at the first dressing. There was a wound in the skin through which the tibia had been thrust. The fracture of the tibia was very oblique, and situated near the upper end of the lower third, while the fracture of the fibula was about two inches below its head.

A roller was applied to the limb and over this pasteboard splints, then another roller and outside of this two lateral wooden splints. The limb was laid carefully on a bed, and properly supported.

Drs. Williams and Allen subsequently had charge of him, and were constant in their attendance, visiting the patient almost every day and renewing the dressings as often as was necessary or useful. After a short time a foot board was attached to a wide side splint, in order to support the foot more effectually.

The splints were continued about five weeks, when the bones were found to be united. In seven weeks he began to use crutches, but he could not bear his weight upon the limb within four months.

During the treatment the knee was always a little flexed, the limb being supported over a double inclined plane of pillows, &c.

One year after, April 17, 1852, McKay called upon me to advise him in relation to a prosecution which he intended to institute against these gentlemen.

The leg is shortened one inch and a quarter, the upper fragment of the tibia overriding the lower, from which it projects about half an inch. The overriding of the fibula can also be distinctly felt. This leg measures half an inch less in circumference than the other. It pains him occasionally at the seat of fracture, and the ankle remains rather stiff.

I believe no suit has ever been instituted, nor do I see upon what part of the treatment a charge of malpractice could be sustained. The treatment is reported as described to me by the complainant himself.

CASE CXXX.—*Simple, oblique fracture of the tibia about six inches above the ankle (middle third,) and simple fracture of the fibula in its upper third. Result imperfect. Union delayed.*

Robert Smith, of Mill Creek, Pa., æt. 46, fell while scuffling, and broke his left leg. Tibia broken six inches from ankle, and obliquely; fibula broken near the upper end.

Robert Faulkner, Homœopathist, was called. Two men made extension, and Faulkner then applied two shingles padded with cotton, and bound them on tightly. On the following day another gentleman, who was formerly a doctor, but now a homœopathist, took charge of the patient with Faulkner. These gentlemen applied an extending and counter-extending apparatus: the counter-extension being made fast below the knee. They drew it very tight. No side splints. On about the eighth day, side splints were added, made of pasteboard. On the twenty-fourth day, all the dressings were removed. On the thirty-first day, a starch bandage was applied, which was continued one week; but becoming painful it was removed and the skin was found to have ulcerated over the projecting seat of fracture, and at other points. Extension and counter-extension were then renewed, but discontinued on the same day. Poultices were applied, &c.; also another form of extending apparatus was adopted. At about the end of the seventh week, everything was removed but a couple of pasteboard splints.

Five months after the fracture had occurred, Mr. Smith called upon me, and I found the leg shortened half an inch; the upper fragment of the tibia overriding the lower. The fragments could be made to move slightly upon each other. The leg was swollen and he had not yet been able to bear his weight upon it. For all these results he blamed the gentlemen who had charge of him, alleging that they were not skillful, and that their treatment was generally bad.

I recommended cold bathing, frictions, &c.

